# Working Memory Development and Motor Vehicle Crashes in Young Drivers

**DOI:** 10.1001/jamanetworkopen.2019.11421

**Published:** 2019-09-13

**Authors:** Elizabeth A. Walshe, Flaura K. Winston, Laura M. Betancourt, Atika Khurana, Kristin Arena, Daniel Romer

**Affiliations:** 1 The Annenberg Public Policy Center at the University of Pennsylvania, Philadelphia; 2 The Children’s Hospital of Philadelphia, Philadelphia, Pennsylvania; 3 College of Education, University of Oregon, Eugene

## Abstract

**Importance:**

Adolescent drivers have the highest rate of motor vehicle crashes, and among equally novice drivers, crash risk is inversely age graded. Working memory (WM), crucial to driving hazard awareness, is also age graded, with ongoing development into late adolescence. Variability in WM capacity and growth trajectory positions WM as a candidate crash risk factor for study, clinical screening, and possible preventative intervention.

**Objective:**

To test the association between crashes and differential WM development.

**Design, Setting, and Participants:**

This prospective cohort study used data from a longitudinal cohort of 118 community youth in Philadelphia, Pennsylvania. Working memory and other risk factors were measured annually from age 11 to 13 years (prelicensure, in 2005) to 14 to 16 years (in 2009), and again at 18 to 20 years (in 2013). In 2015, a follow-up survey of driving experience identified 84 participants who had started driving. Latent growth curve modeling was used to examine the association between variability in the baseline (intercept) and developmental trajectory (slope) of WM and the crash outcome.

**Main Outcomes and Measures:**

Self-reported crashes were the primary outcome. Variability in the relative growth of WM development along with traits and behaviors associated with risky driving were assessed.

**Results:**

Of 84 participants (39 [46%] male; mean [SD] age, 20.46 [1.09] years), 25 (29.8%) reported they had been involved in at least 1 crash. Controlling for other crash risk factors, the model indicated that variation in the linear slope of WM growth was inversely associated with reporting a crash (*b* = −6.41; SE = 2.64; *P* = .02). Crashes were also associated with reckless driving behavior (*b* = 0.40; SE = 0.18; *P* = .03). Variation in the intercept of WM was not associated with crashes (*b* = −0.245; SE = 0.67; *P* = .72).

**Conclusions and Relevance:**

The results suggest that a relatively slower WM growth trajectory is associated with young driver crashes. Routine assessment of WM across adolescence may help to identify at-risk teen drivers and opportunities for providing adaptive interventions (eg, driving aids or training) that can address limitations in WM-related skills that are critical for safe driving.

## Key Points

**Question:**

Is variability in working memory development associated with motor vehicle crashes in young drivers?

**Findings:**

In this cohort study of 84 community youth drivers, relative variation in working memory growth (slope)—but not the baseline (intercept)—was inversely associated with reporting a crash when controlling for other crash risk factors.

**Meaning:**

Routine assessment of working memory development across adolescence may help identify teens with a relatively divergent trajectory of working memory growth who may be more likely to be involved in a motor vehicle crash.

## Teaser

This cohort study investigates the association between working memory development in young drivers and motor vehicle crashes.

## Introduction

Driving, a skill commonly acquired during adolescence, is also a potentially dangerous behavior. Motor vehicle crashes are the leading cause of injury and death for adolescents in the United States.^1^ When compared with other age groups, adolescent drivers have the highest rate of crashes, injuries, and mortality.^2^ Poor skills and inexperience explain some of this risk early after licensure: crash rates are highest during the first 6 months of independent driving.^3,4^ However, even among equally novice drivers, crash risk is age graded. At the outset of independent driving, drivers aged approximately 17 years have a higher crash rate than drivers aged approximately 20 years.^3^ Given this age-graded risk, individual variation in development is likely associated with crash risk, but its nature and contribution to this risk is not known.

One possible developmental source of crash risk is working memory (WM),^5^ which is associated with learning and other risk outcomes in adolescents.^6,7^ Working memory has a protracted development into late adolescence,^8,9^ with intraindividual and interindividual variability,^10^ but generally demonstrates a linear growth trajectory during adolescence.^11,12^ This limited-capacity system allows for in-the-moment monitoring, updating, and planning, which are important for decision making, managing complex tasks, and controlling attention in the face of distraction.^13,14^ Given that driving is a complex task requiring attentional and related abilities (important for situational awareness), variability in WM development may contribute to crashes during adolescence. For example, safe driving involves scanning, monitoring, and updating information about the vehicle and environment while managing multiple subtasks (eg, adjusting speed, steering, in-vehicle controls) and distractors (eg, peer passengers and mobile phones).^15,16^ Working memory development may be even more critical among novice drivers who have not yet automated the subtasks of driving. During the learning phase, adequate cognitive capacity is required to develop both the declarative and procedural memory required for safe driving.^17^ Therefore, if some adolescents start driving before their WM capacity has sufficiently developed, they may be at increased risk for crashes for this reason alone.

Indeed, previous studies have shown that in typically developing teens, lower WM capacity is associated with more reckless and inattentive driving, crashes, and poorer performance on simulated driving tasks.^18,19,20,21^ In addition, driving performance deteriorates when young drivers complete a secondary WM task while driving in a simulator, but is less affected in those with a higher baseline WM capacity.^22^ Furthermore, variability in WM has been associated with impulsivity and substance use during adolescence,^23,24^ known risk factors for reckless driving. However, prior studies were cross-sectional, often focused on driving behavior rather than crash outcomes, and rarely controlled for other known risk factors (eg, impulsivity, substance use). To our knowledge, no studies to date have longitudinally examined WM development and its association with crashes by concurrently investigating WM with personality traits and unsafe behaviors.

Young driver policy statements^25^ outline the importance of considering development when pediatricians counsel families about driving, pointing to recommendations for atypical development (eg, attention-deficit/hyperactivity disorder [ADHD]) as a risk factor. To our knowledge, no evidence exists for recommendations around expected individual variation in typical neurocognitive development, including WM capacity, which co-occurs during the period of learning to drive and early licensure. Therefore, this study aims to enhance the scientific foundation for young driver policies with an initial investigation of individual variation in the trajectory of WM development in association with unsafe driving and crash risk. The question that arises for public policy and pediatric care is whether differential development in WM places some adolescents at risk of crashes due to limitations in attention and related processing skills that are critical for safe driving. This study addresses 2 gaps in the field by (1) assessing the independent prospective association of WM development with young driver crashes while (2) controlling for other risk-related traits and behaviors (impulsivity, sensation seeking, reckless driving, and substance use). We do this using data from a longitudinal trajectory study of community youth that measured WM development from early to late adolescence, as well as the associated risk-related traits and behaviors, and examine the association between these factors and crashes reported in a follow-up survey after a year or more of independent driving.

## Methods

### Participants

Participants were recruited from a larger longitudinal cohort of community youth in Philadelphia, Pennsylvania, the Philadelphia Trajectory Study (PTS).^11^ The original longitudinal study enrolled a community sample of 387 youths aged 10 to 12 years who were assessed annually across 5 waves from 2004 to 2010, and again in 2013 and 2014 at the sixth and final wave of testing, retaining 290 participants (aged 18-20 years). We invited all participants retained at wave 6 to take part in a follow-up survey of driving experiences conducted in 2015. One hundred eighteen participants responded and provided written consent. Of these, 84 participants held a driver’s license and were included in the following analysis. As a check for sample bias, the characteristics of the driver sample and the larger PTS sample were compared.

### Study Design

A survey of driving experience was conducted in 2015 as follow-up to the PTS study. Using latent growth curve analysis (LGC), we examined the developmental trajectory of WM across adolescence using waves 2 to 6 (2006-2012) owing to the addition of an object *n*-back task to the WM battery after wave 1. Only 1 participant was missing WM data at wave 3; otherwise, all other variables in the analysis were complete. We examined the association between the latent intercept and slope of the LGC model and crashes at the follow-up, along with other factors of driving behavior, risk-related traits, and behavior. This study was approved by the institutional review board of the Children’s Hospital of Philadelphia. Participants provided written informed consent. This study followed the Strengthening the Reporting of Observational Studies in Epidemiology (STROBE) reporting guideline for cohort studies.

### Measures

#### Longitudinal Measures

The longitudinal PTS measured WM on 4 tasks and a number of risk traits and behaviors, including impulsivity, sensation seeking, delay discounting, and incidences of fighting behavior and substance use dependency. These measures have been described previously.^11^ A principal component analysis using data from wave 5 and 6 for the total sample of 118 participants revealed that the 2-back, Corsi-block tapping, and spatial WM tasks loaded on 1 factor (explaining 46% of the variance, Cronbach α = 0.83), while the backward digit span loaded on a separate factor alone (explaining 15% of the variance). The 2-back, Corsi-block tapping, and spatial WM scores were standardized to derive a composite score of WM at each wave (Cronbach α ranged from 0.67 to 0.73). This score measured the relative standing of each adolescent in the sample at each wave of the study.

A principal component analysis was used to reduce the risk traits and behaviors to common latent factor scores for use in the LGC model. A risk behavior score derived from wave 6 assessments comprised acting without thinking (AWT) impulsivity, sensation seeking, fighting behavior, alcohol use dependence, and marijuana use dependence. This factor was used in the LGC model to represent this set of risk behaviors near the time of independent driving.

#### Driving Survey

This self-report survey included items adapted from the commonly used Driver Behavior Questionnaire^26^ to gauge license experience (eg, type of license, number of years driving), self-reported history of crashes, and how often participants engaged in reckless and unsafe driving behaviors. Reason et al^26^ noted that items contributing to a factor of reckless driving (intentionally deviating from safe driving by speeding and running traffic lights) were strongly associated with crashes.

Nine high-risk (“definite risk to others”) items were selected from the Driver Behavior Questionnaire to measure engagement in intentional reckless driving (eg, running traffic lights, unsafe overtaking, and ignoring speed limits) and driving errors (eg, failing to check mirrors when pulling out and misjudging the speed of an oncoming vehicle). Given trends of increasing mobile phone use while driving, we added 2 additional items to measure engagement in texting and calls while driving. Participants indicated how often they engaged in each behavior using a 6-point scale (1 = never to 6 = nearly all the time). Many of these items were significantly associated (including the additional mobile phone behavior items) and revealed 2 latent components reflecting intentional reckless driving (including mobile phone use) (Cronbach α = 0.81) and driving errors (Cronbach α = 0.60) (for more detail, see the article by Walshe et al^27^).

#### Crash Outcome

Two questions asked whether the participant had ever been involved in a crash as a driver and, if so, how many crashes. The crash history items were recoded into a single binary crash outcome (0 = never; 1 = at least 1 crash).

### Statistical Analysis

**Figure 1.**
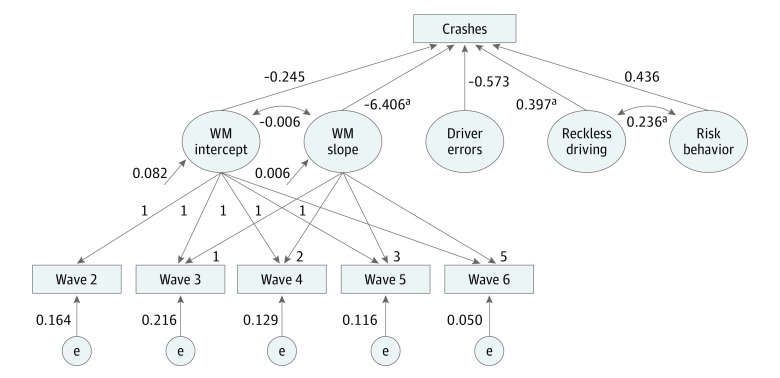
Linear Latent Growth Curve Model Examining the Association Between Working Memory (WM) Development and Crashes Model 1 showing the association between the slope of the WM growth trajectory and crashes. Values at arrows beside latent WM intercept and slope are estimates of variance and values at arrows from factors to crashes indicate parameter estimates. e indicates residual variances of the working memory score at each wave. ^a^Significant parameter values (*P* < .05).

We examined the Pearson correlation coefficients (*r*) between the crash outcome and the individual characteristics recorded at wave 6 of the PTS. We used a LGC model computed in Mplus version 8 (Muthén & Muthén) that was sensitive to the binary crash outcome (using the weighted least-squares estimation method) to examine the association between the baseline (intercept) and developmental trajectories (slope) of WM and crashes (Figure 1). Factor scores for reckless driving, driving errors, and the other risk behaviors described were included in the LGC model (model 1). We then estimated a second model (model 2) with the addition of IQ as a test of robustness. The χ^2^ test statistic, root mean square error of approximation value, comparative fit index, and Tucker-Lewis index were used to determine model fit. Statistical significance was tested at the α = .05 level with 2-tailed tests.

## Results

### Driver Characteristics and Crashes

Table 1 provides the demographic profile of the sample of young drivers. Four of the driver participants had a self-reported ADHD diagnosis, but none of these individuals reported a crash. Of 84 participants, 39 (46%) were male, and the mean (SD) age was 20.46 (1.09) years. The distribution of sex and ethnicity (measured with investigator-defined responses in the PTS study) closely matches that of the rest of the larger PTS study. However, the driver sample was slightly older at wave 6 (driver mean [SD] age, 18.93 [0.77] years; PTS mean [SD] age, 18.61 [0.62] years; difference, 0.32 years; 95% CI, 0.10-0.53 years; *P* = .004), with a slightly higher IQ as measured on the Wechsler Adult Intelligence Scale (driver mean [SD] score, 103.91 [12.13]; PTS mean [SD] score, 98.05 [15.97]; difference, 5.86; 95% CI, 1.54-10.18; *P* = .008). Drivers in the sample had a mean (SD) of 3.46 (1.45) years driving experience, started driving at a mean (SD) age of 17 (1.21) years, and drove for a median (interquartile range) of 4.5 (1-10) hours per week. Twenty-five participants (29.8%) reported that they had been involved in at least 1 crash as a driver, with the majority having only 1 crash (18 had 1 crash, 4 had 2 crashes, and 3 had >2 crashes). The crash outcome was not associated with number of years driving (*r* = 0.21). Many of these young drivers also reported engaging in reckless driving behaviors as captured in the modified Driver Behavior Questionnaire. The most frequently reported reckless behavior was ignoring speed limits (only 32.2% reporting never doing this), and the least reported reckless driving behavior was driving after consuming alcohol (with 85.6% never doing this). Reported crashes were positively associated with reckless driving (*r* = 0.29; *P* = .008), evidencing good construct validity for this crash outcome.

**Table 1.** Demographic Profile of Young Driver Sample

Variable	All Drivers (N = 84)	No Crash (n = 59)	Crash (n = 25)
Age, mean (SD), y	20.46 (1.09)	20.34 (1.07)	20.73 (1.11)
Male, No. (%)	39 (46)	28 (47)	11 (44)
Attention-deficit/hyperactivity disorder, No. (%)	4 (0.05)	4 (0.07)	0
IQ, mean (SD)	104 (12.13)	105 (12.77)	103 (10.56)
Race/ethnicity, No. (%)			
White	53 (63)	44 (75)	17 (68)
African American	13 (15)	7 (12)	6 (24)
Asian	8 (10)	7 (12)	2 (8)
Hispanic	9 (11)	5 (8)	4 (16)
Native American	1 (1)	1 (2)	0

### Correlation Analysis

Table 2 presents the Philadelphia trajectory study measure scores recorded at wave 6 and Table 3 presents the correlations between the individual characteristics recorded at wave 6, the driving behavior factors, and crash outcome measured at follow-up. While reckless driving was correlated with crashes (*r* = 0.29), the driving error factor was not (*r* = −0.18). Crashes did not correlate with age (*r* = 0.16), sex (*r* = 0.03), or IQ (*r* = 0.04). On the other hand, WM at wave 6 was significantly negatively correlated with crashes (*r* = −0.32; *P* = .003). No other individual characteristics (ie, behavior, substance use or dependency) were associated with crashes. However, IQ and marijuana use dependence were positively correlated with WM at wave 6, and so alcohol and marijuana dependence were controlled in the models.

**Table 2.** Philadelphia Trajectory Study Measure Values From the Final Wave (Wave 6)

Measures at Wave 6	Mean (SD) [Range]		
	All Drivers (N = 84)	No Crash (n = 59)	Crash (n = 25)
Working memory			
Backward digit span score	9.04 (2.01) [5-15]	9.02 (1.78) [5-13]	9.08 (2.52) [5-15]
Object 2-back % correct	83.18 (5.12) [59-90]	83.85 (4.87) [59-90]	81.60 (5.40) [63-90]
Corsi-block tapping % correct	44 (13) [19-71]	46 (13) [19-46]	39 (13) [19-66]
Spatial working memory errors, No.	11.35 (9.77) [0-52]	11.90 (10.65) [0-52]	10.04 (7.34) [0-29]
Risk traits and behaviors, No.			
Acting without thinking total	2.01 (2.03) [0-6]	1.97 (1.97) [0-6]	2.12 (2.20) [0-6]
Sensation seeking total	9.85 (2.98) [4-16]	9.78 (2.85) [4-15]	10 (3.33) [4-16]
Delay discounting mean	75.59 (22.94) [10-100]	76.33 (23.52) [10-100]	73.87 (23.37) [10-100]
Fighting behavior count	1.36 (1.11) [1-8]	1.41 (1.28) [1-8]	1.24 (0.52) [1-3]
Use dependence criteria score			
Alcohol	0.56 (0.88) [0-3]	0.47 (0.82) [0-3]	0.76 (1.01) [0-3]
Marijuana	0.36 (0.79) [0-3]	0.41 (0.85) [0-3]	0.24 (0.60) [0-2]

**Table 3.** Pearson Correlation Coefficients (*r*) Between the Individual Characteristic Variables Recorded at Wave 6 and the Follow-up Driving Survey Outcomes

Variable	Variable												
	1	2	3	4	5	6	7	8	9	10	11	12	13
1. Age at follow-up	1												
2. Female sex	0.05	1											
3. Years driving	0.58^a^	−0.01	1										
4. IQ	−0.15	0.06	0	1									
5. Acting without thinking	−0.03	−0.22^b^	0.06	−0.22^b^	1								
6. Delay discounting	−0.18	0.16	0.05	0.33^a^	−0.21	1							
7. Sensation seeking	−0.08	−0.36^a^	−0.01	0.04	0.29^a^	−0.05	1						
8. Fighting behavior	−0.08	−0.22^b^	0.05	−0.03	0.31^a^	0.09	0.08	1					
9. Alcohol use dependence	0.08	−0.09	0.22^b^	0.07	0.29^a^	−0.08	0.16	0.19	1				
10. Marijuana use dependence	0.10	−0.19	0.24^b^	0.03	0.19	−0.07	0.29^a^	0.25^b^	0.44^a^	1			
11. Working memory	−0.17	0.01	−0.06	0.33^a^	−0.15	0.09	0.09	0.04	−0.11	0.02	1		
12. Reckless driving	0	−0.15	0.24^b^	−0.09	0.06	−0.04	0.16	0.06	0.28^b^	0.18	0	1	
13. Diving errors	−0.14	−0.13	0	0.12	0.07	0.15	−0.06	−0.04	0.12	0.19	−0.21	0	1
14. Crashes	0.16	0.03	0.21	−0.08	0.04	−0.03	0.03	0.06	0.15	−0.10	−0.32^a^	0.29^a^	−0.18

^a^ *P* < .01.

^b^ *P* < .05.

### Latent Growth Curve Model

**Figure 2.**
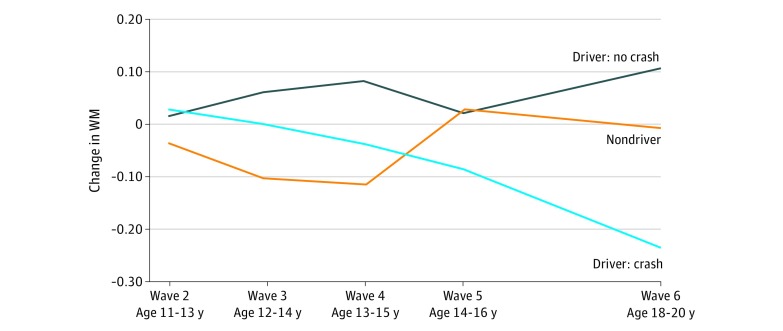
Working Memory (WM) Composite Scores at Each Wave of Assessment Relative changes in working memory from wave 2 to wave 6 assessment points for drivers with a crash history (n = 25), drivers with no crash history (n = 59), and nondrivers who responded to the follow-up survey (n = 34).

We regressed the binary crash outcome on the latent intercept and slope of WM across waves 2 to 6 obtained from the LGC analysis, as well as the factor scores for reckless driving, driving errors, and risk behaviors (model 1). This model provided a good fit to the data (χ^2^_27_ = 25.877; *P* = .53; comparative fit index = 1.00; Tucker-Lewis index = 1.02; and root mean square error of approximation = 0.00 [90% CI, 0.00-0.08]). The model indicated that variation in the slope of the WM growth trajectory had a strong independent negative association with crashes (*b* = −6.41; SE = 2.64; *P* = .02). In addition, reckless driving was also associated with crashes (*b* = 0.40; SE = 0.18; *P* = .03). However, variation in the intercept of WM was not associated with crashes (*b* = −0.245; SE = 0.67; *P* = .72), the driving error factor (*b* = −0.57; SE = 0.33; *P* = .08), or the risk behavior factor (*b* = 0.44; SE = 0.31; *P* = .16) (Figure 1). Among these factors, there was a negative association between the intercept and slope and a positive association between risk behavior and reckless driving; no other factors were significantly correlated. The graph in Figure 2 plots the WM composite scores at each wave, illustrating the relative WM growth trajectories among drivers with and without a crash, as well as nondrivers. Although all 3 groups experienced absolute gains in WM over time, drivers who crashed demonstrated a relatively declining linear trajectory of WM growth that progressively diverged from the other 2 groups, resulting in a larger difference between the groups at wave 6 than at the intercept.

Model 2 included IQ as a possible confounding factor and also provided a good fit to the data (χ^2^_32_ = 36.556; *P* = .27; comparative fit index = 0.95; Tucker-Lewis index = 0.93; root mean square error of approximation = 0.04 [90% CI, 0.00-0.09]). Variation in the slope of WM trajectory (*b* = −7.65; SE = 3.86; *P* = .047) and reckless driving (*b* = 0.46; SE = 0.17; *P* = .008) remained significantly associated with crashes. None of the other variables were significantly associated with crashes, including IQ (*b* = 0.02; SE = 0.02; *P* = .33).

## Discussion

To our knowledge, this is the first longitudinal study of adolescent WM development in association with motor vehicle crashes. The results demonstrated that the variance in the relative trajectory of WM growth (slope), but not the baseline (intercepts), was associated with crashes within approximately 3 years after the start of driving: adolescents with a divergent trajectory of WM growth had a greater likelihood of being involved in a crash. The relative variance in the growth trajectory of WM remained strongly and independently associated with crashes despite controlling for other risk-related factors, such as reckless driving, drug use, and other risk-related traits and behaviors. Furthermore, the model remained robust with the addition of IQ.

These initial longitudinal results coincide with and expand upon previous cross-sectional studies of association between WM and driving behavior in both self-report and simulated driving studies^18,19,20,21^ and may support the argument that insufficient development of WM at the time of independent driving (rather than a stable individual difference) is critical for identifying young drivers at risk for crashes. As such, the rate of WM development may be an important underlying mechanism of age-graded risk for crashes during adolescent development. However, we do not yet know whether or how WM development may predict crashes and need to further investigate factors that lead to differential trajectories of growth in WM to identify high-risk groups.

Future studies should also investigate the role of WM development in the observed increased risk for unsafe driving and crashes among atypically developing populations (eg, ADHD). The study by Curry et al^28^ demonstrated that crash risk is 1.36 times higher among adolescents with a diagnosis of ADHD.^28^ Furthermore, adolescent drivers with ADHD and autism spectrum disorder have more unsafe driving behaviors and errors.^29,30^ Of note, at wave 6 of this study, WM and crashes did not correlate with impulsivity, suggesting that the association between WM and crashes was not mediated by poor impulse control in this community sample. Previous work has shown an inverse association between WM and impulsivity at earlier ages,^23^ but impulsivity does not appear to be associated with later adolescent problem behaviors as well as early behaviors.^31,32^ Future studies with larger sample sizes can better model the effects of impulsivity and substance use, along with WM development, on crash risk. Furthermore, examining clinical populations with impaired impulse control and more severe crash risk may give us insight into how WM development and impulsivity explain crash risk during adolescent development.

### Limitations

There are several limitations of this study. First, the crash outcome was self-reported. However, the finding that reckless driving behavior was associated with crashes is consistent with previous work^26^ and provides some convergent validity for this crash outcome in this study. The crash outcome was not associated with number of years driving, which may be attributed to the fact crash rates are highest within the first year of licensure and our outcome was a cumulative measure of crashes. Second, the sample size may have limited the power to detect associations among the individual characteristic variables (eg, WM development and risk traits and behaviors). Future studies with more power can tease apart the relationships between these factors and the outcome, as well as consider group-based trajectory modeling and latent class analysis to further examine how different developmental trajectories of WM might exert differential influences on crash rates (in both typically and atypically developing adolescents). The association of IQ with WM should also be further examined, with additional factors such as driver training and experience that will change over time with WM development. In addition, while we used a composite score of WM from a battery of performance-based tasks, these measures did not include real-life scenarios or tasks, and so may not be generalizable to how WM would be used during driving. Furthermore, it can be argued that these results could be affected by a practice effect for better learners across waves. However, we do not believe this is likely given that there was at least a year interval between assessments.

## Conclusions

This study builds on the scientific foundation for evidence-based guidelines for the counseling and management of young drivers by addressing individual variation in development. While crash statistics indicate that risk is high in young drivers, most young drivers do not crash. Substantial individual variability exists in neural and cognitive development and patterns of risk in adolescence.^10,33^ Monitoring WM development across adolescence as part of routine assessment could help to identify at-risk drivers, as well as opportunities for intervention. Attention and driving skill deficits due to insufficient WM may be one of the most modifiable risk factors—via experience and skill training. However, we do not yet know how variability in WM contributes to young driver crashes. The advancement of simulated driving technology offers an opportunity to examine how WM development affects skill learning, in-the-moment performance, and risk taking while driving.^5^ Encouragingly, these findings suggest that any interventions that could improve or enhance WM development during adolescence may offer a novel way to reduce the risk outcomes. By identifying the underlying mechanisms of crashes that span typical and atypical development, we can attempt to develop more broadly applicable interventions across multiple populations.
